# New Phytol Derivatives with Increased Cosmeceutical Potential

**DOI:** 10.3390/molecules29204917

**Published:** 2024-10-17

**Authors:** Gonçalo P. Rosa, Ana M. L. Seca, Diana. C. G. A. Pinto, M. Carmo Barreto

**Affiliations:** 1 LAQV-REQUIMTE, Department of Chemistry, University of Aveiro, Campus de Santiago, 3810-193 Aveiro, Portugal; goncalo.p.rosa@uac.pt (G.P.R.); diana@ua.pt (D.C.G.A.P.); 2 University of the Azores, Faculty of Sciences and Technology, cE3c- Centre for Ecology, Evolution and Environmental Changes, Azorean Biodiversity Group, CHANGE–Global Change and Sustainability Institute, 9500-321 Ponta Delgada, Portugal ; maria.cr.barreto@uac.pt

**Keywords:** anti-aging activity, cosmeceutical potential, structural modifications, phytol derivatives, tyrosinase inhibition

## Abstract

Natural compounds are widely incorporated into cosmetic products for many purposes. Diterpenes often function as fragrances, enhancing the sensory experience of these formulations. However, current trends in cosmetic science aim to develop multifunctional products, where compounds traditionally used for texture or fragrance also possess biological activities that contribute to the product’s efficacy. In this context, this study focuses on synthesizing derivatives of phytol—a compound already presents in cosmetic formulations—to enhance its anti-aging properties. The derivatives were synthesized through esterification with substituted benzoic and cinnamic acids, known for their antioxidant and enzyme inhibition properties. Reaction yields ranged from 91.0% to 5.2%, depending on the substituents in acid derivatives. The structures of the synthesized compounds were confirmed through NMR and MS techniques. Both the natural and newly synthesized derivatives were evaluated for their cosmeceutical potential using antioxidant assays and inhibition assays for tyrosinase, elastase, collagenase, and hyaluronidase. This work presents the first report of the synthesis and cosmetic evaluation of several of these derivatives. Comparing with phytol (**1**), which presented an IC_50_ of 77.47 µM, four of the derivatives presented improved tyrosinase inhibitory activity, with phytyl 4-methoxybenzoate being the most active (IC_50_ = 27.9 µM), followed by phytyl benzoate with an IC_50_ of 34.73 µM. Substitutions at other positions on the aromatic ring were less effective. Molecular docking studies confirmed that the modifications potentiated a stronger interaction between the synthesized compounds and tyrosinase.

## 1. Introduction

Natural compounds are invaluable resources for the development of cosmeceutical ingredients, offering potential solutions for skincare, anti-aging treatments, and overall skin health [1]. These compounds, derived from plants, marine organisms, and other natural sources, have been used for centuries in traditional medicine and cosmetics. Their appeal lies not only in their bioactivity but also in their natural origin, which aligns with the increasing consumer demand for clean and sustainable beauty products [2]. However, despite their many benefits, natural compounds often face several challenges, such as low biological activity, stability issues, and unfavorable skin absorption profiles, which can limit their direct application in cosmetic formulations. For instance, some compounds may degrade rapidly when exposed to light or oxygen, reducing their effectiveness in topical products [3]. Additionally, their inability to penetrate the skin barrier efficiently may hinder their ability to act on deeper layers of the skin, where many processes leading to skin aging occurs.

To overcome these challenges, the structural modification of natural products has emerged as a promising strategy [4]. By modifying the scaffolds and functional groups of these compounds, it is possible to enhance their bioactivities, improve skin penetration, and increase their stability under various environmental conditions such as pH, temperature, and light exposure [5]. This approach not only optimizes the efficacy of natural compounds in skincare products but also accelerates the discovery of new, more effective ingredients, capable of addressing specific cosmetic concerns, such as skin hydration, pigmentation control, wrinkle reduction, and protection against oxidative stress [6]. Moreover, chemical modifications can also improve the bioavailability of these compounds, ensuring that they reach their target sites in the skin and maintain their activity over time [7]. Such advances are critical for creating long-lasting and high-performance skincare products that meet the demands of modern consumers.

Phytol (3,7,11,15-tetramethylhexadec-2-en-1-ol), an acyclic monounsaturated diterpene alcohol derived from chlorophyll, is a notable example of the vast potential of natural compounds in both medicinal and cosmetic applications [8]. Phytol is an integral part of the chlorophyll molecule found abundantly in nature and is produced by all photosynthetic organisms, including algae, plants, and cyanobacteria [9]. It also serves as a key metabolite during the breakdown of chlorophyll in ruminant animals, which makes it the most abundant acyclic isoprenoid in the biosphere [8]. Phytol represents an underexplored resource with tremendous potential for cosmetic innovations.

Beyond its well-known role in nutrition—phytol is a precursor to essential nutrients like vitamin K and E—it exhibits a wide range of biological activities. Studies have shown that phytol possesses antioxidant, anti-inflammatory, immunostimulant, and metabolism-modulating effects [8]. Furthermore, phytol and its derivatives have demonstrated a broad spectrum of bioactivities, including antianxiety, cytotoxic, autophagy- and apoptosis-inducing, anti-nociceptive, immune-modulating, and antimicrobial effects [8,10,11,12,13,14]. These properties have raised interest in its potential pharmaceutical and biotechnological applications, particularly in developing treatments for chronic diseases such as cancer and diabetes [15,16,17]. It also reduces oxidative stress [8,18], which makes it a valuable ingredient in antiaging formulations, as it can help to protect skin cells from environmental aggressors like UV radiation and pollution, both of which accelerate the formation of wrinkles and fine lines [19].

The abundance of phytol in nature, combined with its diverse bioactivities, positions it as a commercially important compound in the cosmetic industry, where natural ingredients with proven efficacy are highly sought after.

Given the rising demand for targeted and effective cosmeceutical solutions, the synthesis of phytol derivatives with enhanced cosmeceutical potential is an interesting way of achieving that goal. While previous studies have primarily focused on phytol derivatives with increased antimalarial activity or with increased effectiveness as drug resistance reversal agents [20,21], there has been little research on the impact of chemical modifications to the phytol molecule on its cosmeceutical properties. This represents a significant gap in the literature, as chemically modified derivatives of phytol may enhance their anti-aging activities.

Therefore, this study aims to fill this gap by synthesizing phytol derivatives with increased cosmeceutical potential, specifically focusing on enhancing antioxidant and anti-hyperpigmentation activities, as well as the ability of these phytol derivatives to inhibit extracellular matrix-degrading enzymes such as collagenase, hyaluronidase, and elastase.

## 2. Results and Discussion

### 2.1. Chemistry

**Scheme 1:**
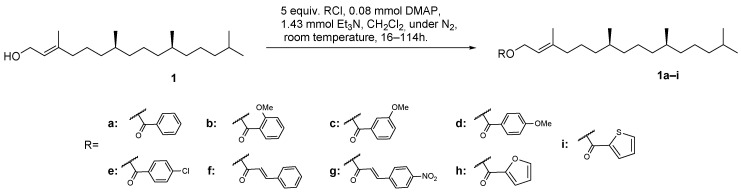
Synthetic route for obtaining derivatives **1a**–**i**.

For the semi-synthesis of phytol derivatives, the primary allylic hydroxy group of phytol (**1**) reacted with various acyl chlorides at room temperature and under a N_2_ atmosphere to obtain derivatives **1a**–**i**, in the conditions shown in Scheme 1.

A total of nine phytol derivatives were synthesized, most of them with yields higher than 85%, showing good efficiency of the chosen method on obtaining the ester derivatives. A summary of the yields is provided in Table 1.

To confirm the successful formation of the desired product, the structure of the compounds obtained was elucidated by NMR and MS and compared to the spectra of the starting compound.

The ^1^H-NMR spectrum of phytol (**1**) showed an olefinic signal at 5.38 ppm, oxymethylene protons at δ 4.11 ppm and signals correspondent to five methyl groups at δ 1.6, 0.84 (6H), and 0.85 (6H) ppm. The ^13^C-NMR spectrum showed the signals of twenty carbons including two olefinic carbons at δ 139.7 and 123.3 ppm, an oxymethylene group signal at δ 59.4 ppm and five methyl groups at δ 22.7, 22.6, 19.7, 19.7, and 16.1 ppm. The remaining signals correspond to nine methylene carbons and four methine carbons as shown on Table 2.

**Figure 1:**
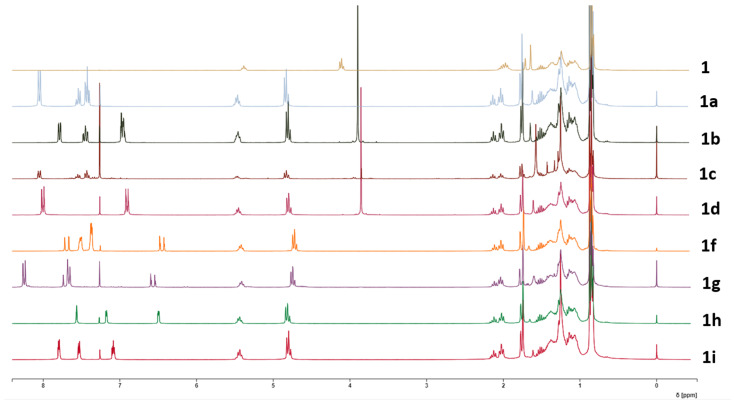
^1^H-NMR spectra of phytol (**1**) and derivatives **1a**–**i**.

The comparison of all the ^1^H-NMR spectra of compounds **1a–i** with that of **1** is depicted in Figure 1.

The ^1^H-NMR spectrum of compound **1a** shows a peak at 4.83 ppm assigned to the methylene protons of C-1. Compared to phytol (4.11 ppm), the chemical shift increased, indicating that the OH group bonded to C-1 is now forming an ester bond. Additionally, the olefinic signal suffered an increase from 5.38 ppm on phytol (**1**) to 5.51–5.44 ppm on **1a**. Three new peaks appear at 8.06–8.03 ppm, 7.61–7.52 ppm, and 7.46–7.36 ppm, which correspond to aromatic protons of a benzene ring. Changes in the ^13^C-NMR spectra correspond to the appearance of the signal of C=O at 166.7 ppm, and at 132.8, 130.6, 129.6, and 128.3 ppm, corresponding to the carbons of the aromatic ring.

The ^1^H-NMR spectra of **1b** showed signals at 7.78–7.75 (dd, 1H), 7.48–7.41 (td, 1H), and 6.99–6.94 (m, 2H) ppm, representative of an *ortho*-substituted aromatic ring. A singlet at 3.91 ppm also appeared, representative of a methoxy group’s presence, which, with the signals from the aromatic ring, indicates this is a 2-methoxy-substituted derivative. As observed in **1a**, the shifts in the H-1 protons increased chemical shift compared to the spectra of the starting compound **1**. In the ^13^C-spectra besides the already-mentioned increase in chemical shift of the signal’s correspondent to C-1, new signals appeared, corroborating the success of the reaction. A signal at 56.0 ppm corresponding to the carbon of the OCH_3_ group, the signal of the carbonyl, at 166.8 ppm and the signals of the aromatic ring at 159.1, 133.2, 131.3, 120.9, 120.1, and 112.1 ppm show the presence of 2-methoxybenzoate on the molecule.

The ^1^H-spectroscopic data of **1c** were like those of **1b**, except for the aromatic protons where the signals at 8.06–8.03 (1H), 7.58–7.52 (1H), 7.46–7.41 (1H), and 7.15–7.11 ppm suggest the methoxy group is in the *meta* position. The ^1^H-NMR spectrum of **1d** also resembled those of **1b** and **1c**, except for protons of the aromatic ring. Two aromatic proton peaks at 8.02–7.97 (dd, *J* = 8.7, 1.4 Hz, 2H) and 6.98–6.83 (dd, *J*= 8.7, 1.4 Hz, 2H) ppm suggest that the methoxy group (3.86, 3H) is located at *para*-position. Signals at the ^1^H proton spectrum of compound **1e**, also indicate the presence of a *para*-substituted ring, with signals at 8.01–7.95 (dd, *J* = 8.8, 1.5 Hz, 2H) and 7.42–7.36 (dd, *J* = 8.8, 1.5 Hz, 2H) ppm.

The ^1^H-NMR spectrum of compound **1f** shows two signals at 7.54–7.51 (m, 2H) and 7.40–7.36 (m, 3H) ppm and two doublets at 7.69 and 6.41 ppm, respectively, with a coupling constant of 16.1 Hz, corresponding to two olefinic protons, which are characteristic of a *trans*-cinnamoyl moiety.

The ^1^H-NMR signals of compound **1g** were like those of **1f**, except for aromatic protons at 7.72–7.68 (d, *J* = 8.8 Hz, 2H) ppm characteristic of a *para*-substituted ring. The peak corresponding to this carbon appears at a higher shift on the ^13^C spectrum (147.9 ppm), indicating it is substituted with an electron-withdrawing group, as is the case of NO_2_. This was confirmed in ESI-MS with the peak at *m*/*z* 472 [M + H]^+^, corresponding to a molecular formula of C_29_H_45_NO_4_.

Analyzing the ^1^H-NMR spectrum of **1h**, it is observable the appearance of signals at 7.57 (dd, *J* = 0.8, 1.7 Hz, 1H), 7.17 (dd, *J* = 3.3, 0.8 Hz, 1H), and at 6.51–6.48 (dd. *J* = 1.7, 3.3 Hz, 1H) ppm, signals characteristic of a furan ring. In conjugation with the displacements observed for the signals of H-1, the formation of the expected compound is confirmed. On the other hand, compound **1i** shows peaks on the ^1^H spectrum at 7.80 (dd, *J* = 1.4, 8.7 Hz, 1H), 7.52 (dd, *J* = 1.4, 5.3 Hz, 1H), and 7.09 (dd, *J =* 5.3, 8.7 Hz) ppm, characteristic of the thiophene ring, and so establishes the depicted structure.

The desired phytol derivatives were obtained with excellent yields with the chosen method with acyl chlorides, with two-thirds of the derivatives exceeding 69%. The low reactivity of the hydroxyl group in phytol, in conjunction with steric hindrance derived from the fact that phytol is a long-chain alcohol with a branched structure prevented the occurrence of the esterification process under other experimental conditions, namely, using *N,N*′-dicyclohexylcarbodiimide (DCC) and 4-pyrrolidinepyridine [22] or POCl_3_ [23], proved to be completely ineffective for the esterification of phytol with cinnamic and benzoic acids. Among all the synthesized derivatives, all compounds except **1a**, **1c**, and **1f** were synthesized for the first time in this work, as far it was able to confirm.

### 2.2. Biological Activities

#### 2.2.1. Level of Activity

The cosmeceutical potential of the starting compound (**1**) and respective derivatives (**1a**–**i**) were evaluated by testing their antioxidant activity, their chelating capacity, and the inhibition of enzymes involved in the aging process. The initial screening of the compounds at a concentration of 100 µM showed that all compounds tested had less than 5% of chelating capacity.

**Figure 2:**
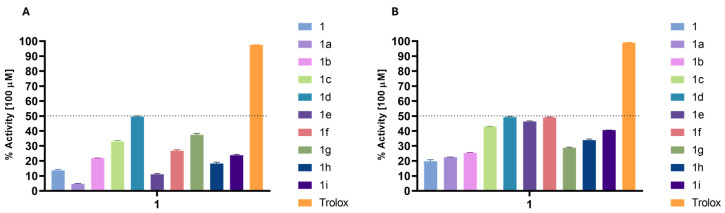
Percentage of DPPH (**A**) and ABTS (**B**) scavenging of the derivatives at a concentration of 100 µM. Trolox was used as antioxidant standard compound.

For the antioxidant activity, the results obtained were significantly more interesting, although the best compounds (**1d** and **1f** derivatives) only reached about 50% of scavenging activity of the DPPH and ABTS radicals (Figure 2), while Trolox, the reference compound, almost reached 90% in both assays at the same concentration.

The results obtained from the DPPH assay generally yielded lower values than those from the ABTS scavenging assay, which was anticipated due to the fundamental differences in the mechanisms of these two assays. ABTS can undergo reduction via hydrogen atom transfer and single electron transfer pathways, accommodating a broader range of antioxidant mechanisms. In contrast, the DPPH assay primarily observes single electron transfer events [24]. Despite the low antioxidant activity shown by all the compounds, there are some noteworthy observations.

In the DPPH assay (Figure 2A), compound (**1d**) exhibited the highest activity (49.55%), indicating that the *para*-OCH_3_ group enhances radical scavenging ability. In contrast **1a** and **1e** show lower activities (4.75% and 11.12%, respectively), suggesting that esterification with benzoic acid or 4-chlorobenzoic reduces antioxidant potential. Derivatives containing OCH_3_ groups generally show higher activity, with a trend favoring substitution at the *para*-position over the *ortho*- or *meta*-positions. Additionally, electron-withdrawing groups like nitro (**1g**, 37.46%) and heterocyclic rings like furan (**1h**) and thiophene (**1i**) also enhance activity, though to a lesser extent. However, none of the derivatives come close to the antioxidant potency of Trolox (97.54%), which serves as the reference standard.

In the ABTS assay, all the derivatives synthesized showed increased activity compared to the respective starting compounds (Figure 2B).

The results show that substitution with 4-methoxy benzoyl (**1d**), cinnamoyl (**1f**), and 4-chloro benzoyl (**1e**) yielded an increase of 30% in antioxidant activity when compared to phytol (**1**). Compound (**1i**) (40.58%) also performed well in ABTS, similarly to its DPPH result. Notably, **1g** shows a lower value on ABTS (28.74%) compared to DPPH (37.46%), indicating differing radical scavenging efficiencies depending on the assay. Overall, the ABTS assay highlights stronger antioxidant responses for 4-methoxylated or chlorinated benzoyl, and cinnamate-based derivatives, with the activities remaining less potent than Trolox across both assays.

**Figure 3:**
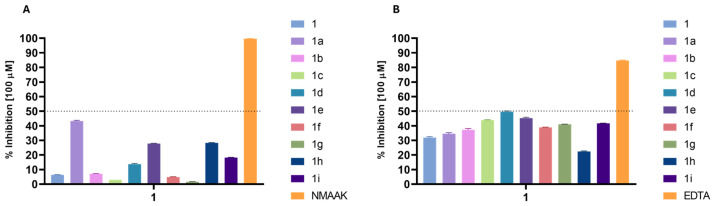
Enzyme inhibitory activity (%) of the derivatives at a concentration of 100 µM: (**A**) elastase inhibition; (**B**) collagenase inhibition. NMAAK: *N*-(methoxysuccinyl)-Ala-Ala-Pro-Val-chloromethyl ketone and EDTA (control inhibitors).

Regarding the inhibition of enzymes related to aging, the results were low for elastase and collagenase inhibition, with none of the compounds reaching 50% inhibition at 100 µM (Figure 3)

The elastase inhibition results (Figure 3A) reveal significant increases in activity for several phytol derivatives compared to phytol itself (6.4%). Compound **1a** stands out with the highest elastase inhibition (43.28%), representing nearly a sevenfold increase in activity over phytol. Phytyl 4-chlorobenzoate (**1e**) and phytyl 2-furoate (**1h**) also show substantial improvements, with inhibition rates of 27.78% and 28.3%, respectively, both showing over fourfold increases in activity compared to phytol. Compound **1i** exhibits an inhibition of 18.19%, which is nearly three times more effective than phytol. Furthermore, when the OCH_3_ substituent group of the benzoyl moiety is on *ortho*- (**1b**) or *meta*- (**1c**) positions, the activity remains low, indicating that these positions need to be free for interaction with the target. These trends highlight that some specific substitutions, particularly benzoate, 4-chlorobenzoate, and furoate moieties, significantly enhance elastase inhibition compared to the parent phytol structure.

The collagenase inhibition results (Figure 3B) show that certain phytol derivatives significantly increase activity compared to phytol (31.89%). Phytyl 4-methoxybenzoate (**1d**) demonstrates the highest inhibition (49.67%), representing a 1.56-fold increase over phytol. Compounds **1c** and **1e** also show improvements (1.42-fold and 1.38-fold, respectively) compared to phytol, indicating better potency when the phytol structure is substituted with a benzoyl group with modifications on *meta*- and *para*-positions. Phytyl 4-nitrocinnamoate (**1g**) and phytyl 2-thiophenecarbonoate (**1i**) also show higher inhibition rates (41.02% and 41.68%, respectively), marking 1.28-fold increases. Phytyl 2-furoate (**1h**), however, shows reduced activity (22.37%), indicating this substitution does not enhance collagenase inhibition. The best results were generally obtained with substituents **d** or **i**, indicating that their conformations must favor the interaction with the enzyme. Since these compounds have not exhibited chelating capacity, their mode of action does not compare with EDTA, which quenches the metallic cofactor of collagenase and consequently inhibits its action.

**Figure 4:**
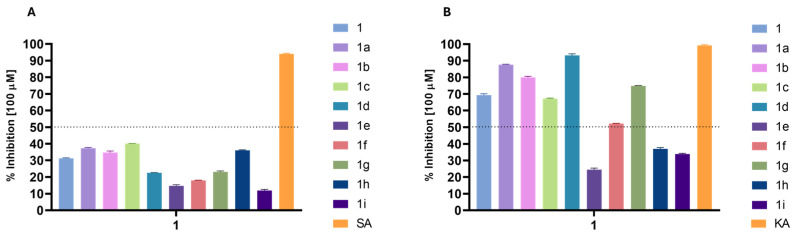
Enzyme inhibitory activity (%) of the derivatives at a concentration of 100 µM: (**A**) hyaluronidase inhibition; (**B**) tyrosinase inhibition. SA: sodium aurothiomalate; KA: kojic acid (control inhibitors).

The hyaluronidase inhibition data (Figure 4A) reveals that phytyl 3-methoxybenzoate (**1c**) shows the highest inhibition at 40.1%, representing a 28% increase over phytol. Compounds **1a** and **1h** also demonstrate improved inhibition (37.32% and 36.02%, respectively), with both increasing activity by about 15–20%. Phytyl 2-methoxybenzoate (**1b**) shows a slight enhancement (34.74%), representing a modest increase over phytol. On the other hand, some derivatives, such as **1d**, **1e**, and **1g**, have reduced inhibition rates (22.54%, 14.66%, and 23.06%, respectively), indicating that both benzoyl and cinnamoyl moieties must be unprotected at *para* position to interact with the enzyme. Overall, *ortho*- or *meta*-methoxybenzoyl and furoyl substitutions provide the most substantial increases in anti-hyaluronidase activity.

The results were more promising for tyrosinase inhibition (Figure 4B), with some of the compounds presenting a high percentage of inhibition, which allowed for calculating their IC_50_ (Table 3).

As observed in Table 3, some modifications led to a significant increase in inhibitory activity against tyrosinase. Compound **1a** displayed an IC_50_ of 34.73 ± 0.10 µM, making it 2.2 times more potent than the parent compound **1** (77.47 ± 0.80 µM). This modification highlights that the introduction of a benzoate group substantially enhances activity. A further improvement was observed with compound **1d** (27.94 ± 0.63 µM), which contains a 4-methoxybenzoate substitution, yielding the most potent activity in this series, representing a significant improvement compared to the starting compound **1**, even though it remains 2.27 times less potent than the reference inhibitor, kojic acid (12.30 ± 0.10 µM). This suggests that the *para* OCH_3_ substitution offers beneficial electronic effects, contributing to increased tyrosinase inhibition. Interestingly, the activity of the *ortho*- (**1b**, 59.62 ± 0.51 µM) and *meta*- (**1c**, 78.96 ± 0.87 µM) methoxy-substituted derivatives was lower, which could indicate that steric hindrance or less favorable electronic interactions in these positions reduce the inhibitory potential. This trend suggests that substitution patterns significantly impact activity.

In contrast, compounds **1e**, **1h**, and **1i** exhibited very weak or no activity (Figure 4B), with IC_50_ values of >100 µM, showing that substituents like 4-chlorobenzoate and heterocyclic groups such as 2-furoyl and 2-thiophenecarbonyl interfere with effective tyrosinase inhibition. Furthermore, compound **1f** also demonstrated weak activity (95.68 ± 0.94 µM), reinforcing the idea that specific substitutions may negatively affect enzyme interaction.

#### 2.2.2. Mode of Inhibition

**Figure 5:**
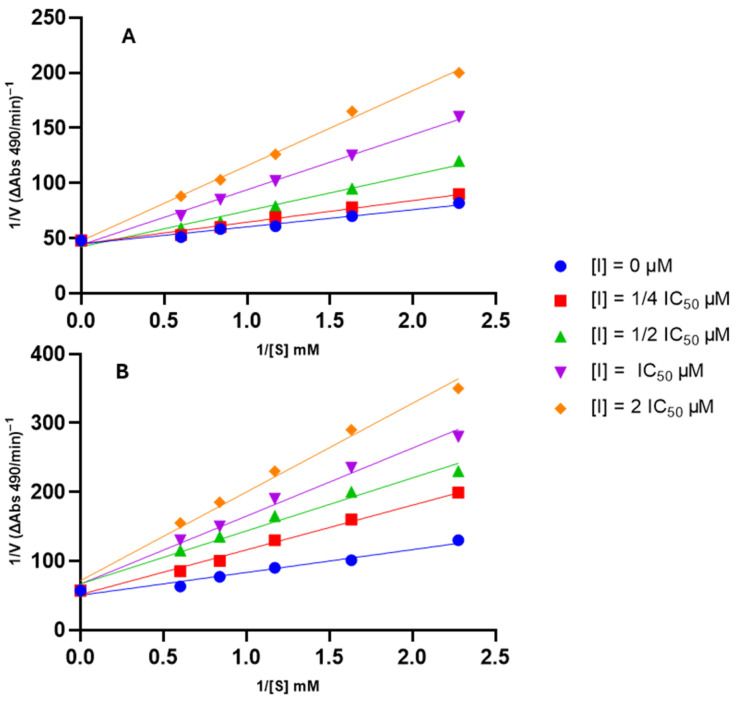
Lineweaver–Burk plots for type of inhibition of tyrosinase by the tested compounds. Panel (**A**): compound **1**; Panel (**B**): compound **1d**.

The mode of inhibition of the compound exhibiting the highest anti-tyrosinase activity, **1d**, was elucidated by assessing its effect on K_m_ (dissociation constant) and V_max_ (maximum reaction velocity) values using a Lineweaver–Burk plot (Figure 5). This analysis was also conducted for the starting compound, phytol (**1**), to understand whether the modifications affected the mode of inhibition.

As depicted in Figure 5, each compound yielded a plot where curves with varying slopes (each corresponding to a different inhibitor concentration) intersected the y-axis almost at the same point. This result indicates that V_max_ values remained unaffected by inhibitor concentration, while K_m_ values exhibited a gradual increase with rising inhibitor concentration. These observations suggest that the compounds are competitive inhibitors of tyrosinase.

#### 2.2.3. Molecular Docking

Molecular docking simulations were performed to study the interactions between the derivatives synthesized and the targets tested on the enzyme inhibition assays where the best results were obtained, namely, tyrosinase and hyaluronidase. Studying these interactions allows for a better understanding of the possible mechanisms of action and an assessment of the effects of different substituents on the activity of the compounds.

Since compound **1d** presented the highest activity against tyrosinase, it was selected for molecular docking simulations. Compound **1**, being the starting compound, was also included to compare the effects of the addition of substituents on the interaction with the target. Compound **1a** was also included for possessing the unsubstituted benzoyl moiety, as well as **1b**–**1c**, which possess the methoxy substitution on a different position in the ring and presented lower activities than **1d**.

For each simulation, a single ligand is docked individually with the protein. The resulting scores and poses are then compared to select the optimal pose, with the understanding that the lowest score indicates the best binding affinity. The results obtained are summarized in Table 4.

The binding results concerning tyrosinase mirror the outcomes of the in vitro assay, as the compounds with higher affinity for tyrosinase also demonstrated stronger inhibitory activity against the enzyme. All tested ligands interacted with residues in the catalytic center, suggesting that they exert their inhibitory effects by competing with the substrate. This confirms the experimental data for compounds **1** and **1d**, which are reported in Section 2.2.1.

Compounds **1a**–**1d** all interact with the His263 and Met280 residues, corresponding to the interaction of the phytol backbone and kojic acid with the enzyme. The addition of a benzoyl moiety to phytol (**1a**) leads to interactions with His65 and Arg268, further increasing its affinity for the catalytic site of tyrosinase. The introduction of a 2-methoxybenzoyl group (**1b**) disrupts the interaction with the His65 residue. This effect extends to His85 when the substituent is 3-methoxybenzoyl (**1c**), resulting in a loss of affinity and a corresponding decrease in the compound’s potency. However, when the derivative has the methoxy group in the *meta*-position (**1d**), the affinity is significantly increased, with the ligand interacting with a greater number of residues; three of them (His85, His263, and Met280) are the same residues with which Kojic acid interacts. Additionally, the literature indicates that protocatechuic acid, a strong natural tyrosinase inhibitor, exhibits a great affinity for the active center of the enzyme (binding score −6.3 Kcal/mol), interacting with residues His85, His259, Asn260, Phe264, and His263 [25], having in common several residues with **1d** and kojic acid. These similarities may indicate that these compounds exhibit similar mechanisms of action.

The fact that all compounds **1**–**1d** exhibit lower activity and a lower binding score than Kojic acid, and simultaneously they all interact with the residue Arg268, contrary to what happens with kojic acid, seems to indicate that this interaction causes less inhibition of enzyme active center.

Given the excellent anti-aging properties exhibited by some of the synthesized derivatives, especially compound 1d, it is necessary to reflect on the challenges that must be considered when introducing this compound into a new formulation. Due to the hydrophobic nature of this compound, it presents a low solubility in aqueous media, which will imply careful attention to the oil/water ratio of the formulation and consequent stability, with no significant difficulties expected regarding the penetration capacity of the formulation, nor in the choice of emollients.

## 3. Materials and Methods

### 3.1. General

The ^1^H- and ^13^C-NMR, HSQC, HMBC, DEPT, COSY, and H2BC spectra were measured on Bruker Avance 300 (Wissembourg, France) (300.13 MHz for ^1^H and 75.47 MHz for ^13^C, respectively), always using TMS as internal standard. Chemical shifts were reported in δ units (ppm) and coupling constants (*J*) in Hz. The MS spectra were obtained using ESI (+) with a Q-Tof_2_ mass spectrometer (Manchester, UK).

Reactions were routinely monitored by thin-layer chromatography (TLC) in silica gel 60 (Merck F_254_ plates) (Darmstadt, Germany) and the products visualized with ultraviolet lamp (254 and/or 365 nm). The chromatographic purifications were carried out by preparative TLC under the same conditions (silica gel 60 (Merck F_254_ plates).

### 3.2. Standards and Reagents

Commercially available *trans*-phytol (97%) was used without any previous purification and was obtained from Sigma Aldrich (San Louis, MO, USA). The solvents used during both reactions and purification procedures were analytically pure or were, if necessary, dried using the appropriate molecular sieves of 3Å.

1,1-diphenyl-2-picryl-hydrazyl (DPPH), 6-hydroxy-2,5,7,8-tetramethylchroman-2-carboxylic acid (Trolox), 2,2′-azinobis-(3-ethylbenzothiazoline-6-sulfonic acid)(ABTS), potassium persulfate, tyrosinase, L-tyrosine, kojic acid, monosodium phosphate, sodium phosphate dibasic, elastase, *N*-methoxysuccinyl-Ala-Ala-Pro-Val-*p*-nitroanilide, *N*-methoxysuccinyl-Ala-Ala-Pro-chloromethylketone, 2-[4-(2-hydroxyethyl)piperazin-1-yl]ethanesulfonic acid (HEPES), *N*-[3-(2-Furyl)acryloyl]-Leu-Gly-Pro-Ala (FALGPA), *N*-[Tris(hydroxymethyl)methyl]-2-aminoethanesulfonic acid (TES) sodium salt, ninhydrin, citric acid, sodium citrate, EDTA, hyaluronidase, hyaluronic acid, calcium chloride, sodium aurothiomalate, sodium acetate, acetic acid and hydrochloric acid, dimethylsulfoxide (DMSO), dichloromethane, chloroform, and ethyl acetate were supplied by Sigma-Aldrich. The collagenase was supplied by Merck. Tin (II) chloride and methanol were obtained from Honeywell Riedel-deHaën (Seelze, Germany).

### 3.3. Synthesis of Derivatives

To a solution of phytol (**1)** at 0.012–0.29 mmol in 10 mL of dichloromethane, 5 equivalents of the appropriate acyl chloride, 1.43 mmol of Et_3_N, and 0.08 mmol of DMAP were added. The mixture was agitated at room temperature under N_2_ atmosphere for 16–114 h. The reaction was then terminated by diluting the solution with 10 mL of dichloromethane and washing with 10 mL of saturated solutions of NaHCO_3_ and NaCl. The organic phase was dried over anhydrous NaSO_4_, filtered, and dried on the rotavapor. The filtrate was purified by thin layer chromatography to give the expected products **1a**–**1i**.

**Phytol (starting material)**: Viscous liquid. ^1^H-NMR (300.13 MHz, CDCl_3_, TMS) and ^13^C-NMR (75.47 MHz, CDCl_3_, TMS) see Table 2.

**Phytyl benzoate (1a):** Viscous liquid; TLC: hexane/ethyl acetate 9:1; yield: 71.6 mg, 86.6%; ^1^H-NMR (300.13 MHz, CDCl_3_, TMS): δ_H_ 8.06–8.03 (2H, dd, *J* = 8.5, 1.4 Hz, H-2′, H-6′), 7.61–7.52 (1H, ddd, *J* = 8.5, 7.4, 1.4, H-4′), 7.46–7.41 (2H, m, H-3′, H5′), 5.51–5.44 (1H, m, H-2); 4.82 (2H, t, *J* = 7.5 Hz, H-1), 2.05–1.92 (2H, m, H-4), 1.65 (3H, s, CH_3_-20), 0.85 (6H, d, *J* = 8.5 Hz, CH_3_–18, CH_3_-19), 0.84 (6H, d, *J* = 8.5Hz, CH_3_-16, CH_3_-17) ppm;

^13^C-NMR (75.47 MHz, CDCl_3_, TMS): δ_C_ 166.0 (C=O), 139.7 (C-3), 132.8 (C-4′), 129.6 (C-2′, C6′), 128.2 (C-3′, C5′), 120.1 (C-1′), 118.3 (C-2), 61.9 (C-1), 39.9 (C-4), 39.4 (C-14), 37.4 (C-6), 37.3 (C-8, C-12), 36.7 (C-10), 32.8 (C-11), 32.7 (C-7), 28.0 (C-15), 25.2 (C-2), 24.8 (C-13), 24.5 (C-9), 22.7 (C-17), 22.6 (C-16), 19.7 (C-18, C-19), 16.1 (C-20); ESI-MS: *m*/*z* 401 [M + H]^+^, 423 [M + Na]^+^.

**Phytyl 2-methoxybenzoate (1b):** Viscous liquid; TLC: hexane/ethyl acetate 8:2; yield: 106.9 mg, 91.0 %; ^1^H-NMR (300.13 MHz, CDCl_3_, TMS): δ_H_ 7.78–7.75 (1H, dd, *J* = 7.9, 1.9 Hz, H-6′), 7.48–7.41 (1H, td, *J* = 8.6, 7.5, 1.9, H-4′), 6.99–6.94 (2H, m, H-3′, H5′),5.51–5.44 (1H, m, H-2); 4.83 (2H, t, *J* = 7.5 Hz, H-1), 3.91 (3H, s, 2′-OCH_3_), 2.05–1.92 (2H, m, H-4), 1.64 (3H, s, CH_3_-20), 0.85 (6H, d, *J* = 8.5 Hz, CH_3_-18, CH_3_-19), 0.84 (6H, d, *J* = 8.5Hz, CH_3_-16, CH_3_-17) ppm;

^13^C-NMR (75.47 MHz, CDCl_3_, TMS): δ_C_ 166.8 (C=O), 159.1 (C-2′), 139.7 (C-3), 133.2 (C-4′), 131.3 (C-6′), 120.9 (C-5′), 120.1 (C-1′), 118.3 (C-2), 112.1 (C-3′), 61.9 (C-1), 56.0 (OCH_3_), 39.9 (C-4), 39.4 (C-14), 37.4 (C-6), 37.3 (C-8, C-12), 36.7 (C-10), 32.8 (C-11), 32.7 (C-7), 28.0 (C-15), 25.2 (C-2), 24.8 (C-13), 24.5 (C-9), 22.7 (C-17), 22.6 (C-16), 19.7 (C-18, C-19), 16.1 (C-20). ESI-MS: *m*/*z* 431 [M + H]^+^.

**Phytyl 3-methoxybenzoate (1c):** Viscous liquid; TLC: hexane/ethyl acetate 9:1; yield: 4.8 mg, 5.2 %; ^1^H-NMR (300.13 MHz, CDCl_3_, TMS): δ_H_ 8.06–8.03 (1H, ddd, *J* = 8.6, 1.8, 1.2 Hz, H-6′), 7.58–7.52 (1H, ddd, *J* = 1.8, 1.2, 0.4 Hz, H-2′), 7.46–7.41 (1H, ddd, *J* = 8.6, 8.2, 0.4, H-5′), 7.15–7.11 (1H, ddd, *J=* 8.2, 1.8, 1.2, H-4′), 5.51–5.44 (1H, m, H-2); 4.83 (2H, t, *J* = 7.5 Hz, H-1), 3.90 (3H, s, 3′-OCH_3_), 2.05–1.92 (2H, m, H-4), 1.64 (3H, s, CH_3_-20), 0.85 (6H, d, *J* = 8.6 Hz, CH_3_-18, CH_3_-19), 0.84 (6H, d, *J* = 8.6 Hz, CH_3_-16, CH_3_-17) ppm;

^13^C-NMR (75.47 MHz, CDCl_3_, TMS): δ_C_ 166.9 (C=O), 153.3 (C-3′), 139.7 (C-3), 132.8 (C-2′), 129.6 (C-6′), 128.2 (C-5′), 120.9 (C-4′), 120.1 (C-1′), 118.3 (C-2), 61.9 (C-1), 55.9 (OCH_3_), 39.9 (C-4), 39.4 (C-14), 37.4 (C-6), 37.3 (C-8, C-12), 36.7 (C-10), 32.8 (C-11), 32.7 (C-7), 28.0 (C-15), 25.2 (C-2), 24.8 (C-13), 24.5 (C-9), 22.7 (C-17), 22.6 (C-16), 19.7 (C-18, C-19), 16.1 (C-20); ESI-MS: *m*/*z* 431 [M + H]^+^.

**Phytyl 4-methoxybenzoate (1d):** Viscous liquid; TLC: hexane/ethyl acetate 8:2; yield: 83.3 mg, 79.1 %; ^1^H-NMR (300.13 MHz, CDCl_3_, TMS): δ_H_ 8.02–7.97 (2H, dd, *J* = 8.7, 1.4 Hz, H-2′, H-6′), 6.98–6.83 (2H, dd, *J* = 8.7, 1.4 Hz, H-3′, H-5′), 5.51–5.44 (1H, m, H-2); 4.83 (2H, t, *J* = 7.6 Hz, H-1), 3.86 (3H, s, 4′-OCH_3_), 2.05–1.92 (2H, m, H-4), 1.64 (3H, s, CH_3_-20), 0.85 (6H, d, *J* = 8.5 Hz, CH_3_-18, CH_3_-19), 0.84 (6H, d, *J* = 8.5Hz, CH_3_-16, CH_3_-17) ppm;

^13^C-NMR (75.47 MHz, CDCl_3_, TMS): δ_C_ 167.1 (C=O), 159.8 (C-4′), 139.7 (C-3), 132.1 (C-2′), 131.9 (C-6′), 123.1 (C-1′), 118.3 (C-2), 112.3 (C-5′), 112.1 (C-3′), 61.9 (C-1), 55.8 (OCH_3_), 39.9 (C-4), 39.4 (C-14), 37.4 (C-6), 37.3 (C-8, C-12), 36.7 (C-10), 32.8 (C-11), 32.7 (C-7), 28.0 (C-15), 25.2 (C-2), 24.8 (C-13), 24.5 (C-9), 22.7 (C-17), 22.6 (C-16), 19.7 (C-18, C-19), 16.0 (C-20); ESI-MS: *m*/*z* 431 [M + H]^+^.

**Phytyl 4-chlorobenzoate (1e):** Viscous liquid; TLC: hexane/dichloromethane 8:2; yield: 81.4 mg, 69.3 %; ^1^H-NMR (300.13 MHz, CDCl_3_, TMS): δ_H_ 8.01–7.95 (2H, dd, *J* = 8.8, 1.5 Hz, H-2′, H-6′), 7.42–7.36 (2H, dd, *J* = 8.8, 1.5 Hz, H-3′, H-5′), 5.51–5.44 (1H, m, H-2); 4.83 (2H, t, *J* = 7.5 Hz, H-1), 2.05–1.92 (2H, m, H-4), 1.64 (3H, s, CH_3_-20), 0.85 (6H, d, *J* = 8.5 Hz, CH_3_-18, CH_3_-19), 0.84 (6H, d, *J* = 8.5Hz, CH_3_-16, CH_3_-17) ppm;

^13^C-NMR (75.47 MHz, CDCl_3_, TMS): δ_C_ 166.8 (C=O), 139.7 (C-3), 133.7 (C-4′), 131.1 (C-2′), 129.9 (C-6′), 128.1 (C-1′), 127.1 (C-3′), 126.9 (C-5′), 118.3 (C-2), 61.9 (C-1), 39.9 (C-4), 39.4 (C-14), 37.4 (C-6), 37.3 (C-8, C-12), 36.7 (C-10), 32.8 (C-11), 32.7 (C-7), 28.0 (C-15), 25.2 (C-2), 24.8 (C-13), 24.5 (C-9), 22.7 (C-17), 22.6 (C-16), 19.7 (C-18, C-19), 16.1 (C-20); ESI-MS: *m*/*z* 436 [M + H]^+^.

**Phytyl cinnamoate (1f):** Viscous liquid; TLC: hexane/ethyl acetate 9:1; yield: 76.7 mg, 88.8 %; ^1^H-NMR (300.13 MHz, CDCl_3_, TMS): δ_H_ 7.69 (1H, d, *J* = 16.1 Hz, Hβ), 7.54–7.51 (2H, m, H-2′, H-6′), 7.40–7.36 (3H, m, H-3′, H-4′, H-5′), 6.41 (1H, d, *J* = 16.1 Hz, Hα), 5.51–5.44 (1H, m, H-2); 4.83 (2H, t, *J* = 7.5 Hz, H-1), 2.05–1.92 (2H, m, H-4), 1.64 (3H, s, CH_3_-20), 0.85 (6H, d, *J* = 8.5 Hz, CH_3_-18, CH_3_-19), 0.84 (6H, d, *J* = 8.5Hz, CH_3_-16, CH_3_-17) ppm;

^13^C-NMR (75.47 MHz, CDCl_3_, TMS): δ_C_ 166.9 (C=O), 145.7 (C-β), 139.7 (C-3), 130.4 (C-1′), 128.4 (C-3′), 128.2 (C-5′), 127.9 (C-4′), 127.5 (C-6′), 120.4 (C-α), 118.3 (C-2), 61.9 (C-1), 39.9 (C-4), 39.4 (C-14), 37.4 (C-6), 37.3 (C-8, C-12), 36.7 (C-10), 32.8 (C-11), 32.7 (C-7), 28.0 (C-15), 25.2 (C-2), 24.8 (C-13), 24.5 (C-9), 22.7 (C-17), 22.6 (C-16), 19.7 (C-18, C-19), 16.1 (C-20); ESI-MS: *m*/*z* 436 [M + H]^+^.

**Phytyl 4-nitrocinnamoate (1g):** Viscous liquid; TLC: chloroform; yield: 20.1 mg, 25.1 %; ^1^H-NMR (300.13 MHz, CDCl_3_, TMS): δ_H_ 8.27–8.24 (2H, d, *J* = 8.8 Hz, H-3′, H-5′), 7.68 (1H, d, *J* = 16.1 Hz, Hβ), 7.72–7.68 (2H, d, *J* = 8.8 Hz, H-2′, H-6′), 6.41 (1H, d, *J* = 16.1 Hz, Hα), 5.51–5.44 (1H, m, H-2); 4.83 (2H, t, *J* = 7.5 Hz, H-1), 2.05–1.92 (2H, m, H-4), 1.64 (3H, s, CH_3_-20), 0.85 (6H, d, *J* = 8.5 Hz, CH_3_-18, CH_3_-19), 0.84 (6H, d, *J* = 8.5Hz, CH_3_-16, CH_3_-17).ppm;

^13^C-NMR (75.47 MHz, CDCl_3_, TMS): δ_C_ 169.2 (C=O), 147.9 (C-4′), 145.6 (C-β), 139.7 (C-3), 130.3 (C-1′), 128.6 (C-3′), 128.5 (C-5′), 123.6 (C-2′), 123.3 (C-6′), 120.3 (C-α), 118.3 (C-2), 61.9 (C-1), 39.9 (C-4), 39.4 (C-14), 37.4 (C-6), 37.3 (C-8, C-12), 36.7 (C-10), 32.8 (C-11), 32.7 (C-7), 28.0 (C-15), 25.2 (C-2), 24.8 (C-13), 24.5 (C-9), 22.7 (C-17), 22.6 (C-16), 19.7 (C-18, C-19), 16.1 (C-20); ESI-MS: *m*/*z* 472 [M+H]^+^.

**Phytyl 2-furoate (1h):** Viscous liquid; TLC: hexane/ethyl acetate 8:2; yield: 102.9 mg, 85.0 %; ^1^H-NMR (300.13 MHz, CDCl_3_, TMS): δ_H_ 7.57 (1H, dd, *J* = 0.8, 1.7 Hz, H-3′), 7.17 (1H, dd, *J* = 3.3, 0.8 Hz, H-5′), 6.51–6.48 (1H, dd, *J* = 1.7, 3.3 Hz, H-4′), 5.51–5.44 (1H, m, H-2); 4.83 (2H, t, *J* = 7.5 Hz, H-1), 2.05–1.92 (2H, m, H-4), 1.64 (3H, s, CH_3_-20), 0.85 (6H, d, *J* = 8.5 Hz, CH_3_-18, CH_3_-19), 0.84 (6H, d, *J* = 8.5Hz, CH_3_-16, CH_3_-17) ppm;

^13^C-NMR (75.47 MHz, CDCl_3_, TMS): δ_C_ 166.6 (C=O), 143.9 (C-1′), 143.6 (C-3′), 139.7 (C-3), 119.8 (C-5′),112.1 (C-4′), 118.3 (C-2), 61.9 (C-1), 39.9 (C-4), 39.4 (C-14), 37.4 (C-6), 37.3 (C-8, C-12), 36.7 (C-10), 32.8 (C-11), 32.7 (C-7), 28.0 (C-15), 25.2 (C-2), 24.8 (C-13), 24.5 (C-9), 22.7 (C-17), 22.6 (C-16), 19.7 (C-18, C-19), 16.1 (C-20); ESI-MS: *m*/*z* 391 [M + H]^+^.

**Phytyl 2-thiophenecarbonoate (1i):** Viscous liquid; TLC: hexane/ethyl acetate 8:2; yield: 80.8 mg, 90.3 %; ^1^H-NMR (300.13 MHz, CDCl_3_, TMS): δ_H_ 7.80 (1H, dd, *J* = 1.4, 8.7 Hz, H-3′), 7.52 (1H, dd, *J* = 1.4, 5.3 Hz, H-5′), 7.09 (1H, dd, *J* = 5.3, 8.7 Hz, H-4′), 5.51–5.44 (1H, m, H-2); 4.83 (2H, t, *J* = 7.5 Hz, H-1), 2.05–1.92 (2H, m, H-4), 1.64 (3H, s, CH_3_-20), 0.85 (6H, d, *J* = 8.5 Hz, CH_3_-18, CH_3_-19), 0.84 (6H, d, *J* = 8.5Hz, CH_3_-16, CH_3_-17) ppm;

^13^C-NMR (75.47 MHz, CDCl_3_, TMS): δ_C_ 166.7 (C=O), 139.7 (C-3), 134.7 (C-5′), 131.0 (C-1′), 127.6 (C-4′), 118.4 (C-2), 61.8 (C-1), 39.9 (C-4), 39.4 (C-14), 37.4 (C-6), 37.2 (C-8, C-12), 36.7 (C-10), 32.8 (C-11), 32.7 (C-7), 28.0 (C-15), 25.2 (C-2), 24.8 (C-13), 24.5 (C-9), 22.7 (C-17), 22.6 (C-16), 19.7 (C-18, C-19), 16.1 (C-20); ESI-MS: *m*/*z* 407 [M + H]^+^.

### 3.4. Biological Activities

A stock solution of each sample was prepared at 60 mM in DMSO, from which the test samples were then prepared by diluting in the appropriate test medium. In microplate assays, absorbance values were measured using a BioRad Microplate Reader Model 680 (Bio-Rad Laboratories, Inc., Hercules, CA, USA) at the wavelength indicated below for each test.

#### 3.4.1. DPPH Radical Scavenging Activity

Antioxidant activity was assayed by the 1,1-diphenyl-2-picryl-hydrazyl (DPPH) radical scavenging assay [26]. Serial dilutions of studied extracts or reference compound (Trolox) were carried out in 96-well microplates, with concentrations ranging between 0.100 μM and 100 μM in methanol. DPPH dissolved in methanol was added to the microwells, yielding a final concentration of 45 µg/mL, and the absorbance at 515 nm was measured after 30 min in the dark. In each assay, a control was prepared in which the same amount of solvent substituted the sample or standard. Percentage of antioxidant activity (% AA) was calculated as

%AA = [(Abs_control_ − Abs_sample_)/Abs_control_] × 100 (1)

where Abs_control_ is the absorbance of the control and Abs_sample_ is the absorbance of the alga extract or standard. All assays were carried out in triplicate and the results expressed as IC_50_, i.e., as the concentration yielding 50% scavenging of DPPH, calculated by interpolation from the % AA vs. concentration curve.

#### 3.4.2. ABTS Radical Scavenging Assay

The method of Re et al. [27] was adopted to perform the ABTS radical scavenging assay. The stock solutions included a 7 mM ABTS solution (2,2′-azinobis-(3 ethylbenzothiazoline 6 sulfonic acid)) and a 2.4 mM potassium persulfate solution. The working solution was prepared by mixing the two stock solutions in equal quantities and allowing for them to react for 12–16 h at room temperature in the dark. The solution was then diluted by mixing 1 mL ABTS solution with the amount of methanol necessary to obtain an absorbance of 0.7 at 734 nm. Serial dilutions of studied extracts or reference compound (Trolox) were carried out in 96-well microplates, with concentrations ranging between 0.100 μM and 100 μM in methanol. ABTS solution was then added to the microwells, and after 8 min of incubation, the absorbance was recorded at 750 nm. In each assay, a negative control was prepared in which the same amount of solvent substituted the sample. The percentage of antioxidant activity (% AA) was calculated as

%AA = [(Abs_control_ − Abs_sample_)/Abs_control_] × 100 (2)

where Abs_control_ is the absorbance of ABTS radical + methanol; Abs_sample_ is the absorbance of ABTS radical + sample/standard.

All assays were carried out in triplicate and results expressed as IC_50_, i.e., as the concentration yielding 50% scavenging of ABTS, calculated by interpolation from the % AA vs. concentration curve.

#### 3.4.3. Ferrous Chelating Activity

The Fe^2+^ chelating ability of the extracts was measured by the ferrous iron–ferrozine complex method [28]. Briefly, the reaction mixture containing 2 mM FeCl_2_ (10 µL), 5 mM ferrozine (10 µL), and 100 µL of serial concentrations of extracts or fractions (ranging between 0.10 µM and 100 µM in methanol) were mixed in a 96-well plate and incubated for 10 min at 27 °C. The absorbance was at 550 nm. The absorbance of the control was determined by replacing the extract with methanol. EDTA (0.10–100 µM)) was used as a positive control. The ability of the sample to chelate ferrous ion was calculated as follows:

Chelating effect (%) = [(Abs_control_ − Abs_sample_)/Abs_control_] × 100 (3)

where Abs_control_ is the absorbance of the ferrozine–ferrous iron complex + methanol; Abs_sample_ is the absorbance ferrozine–ferrous iron complex + sample/standard.

All assays were carried out in triplicate and results expressed as IC_50_, i.e., value which was the concentration of the sample that chelated 50% of the ferrous iron, calculated by interpolation from the % chelating effect vs. concentration curve.

#### 3.4.4. Hyaluronidase Inhibition Assay

The assay was conducted following the Sigma protocol with minor adjustments [29]. In a microplate, 25 µL of 5 U/mL of hyaluronidase prepared in 20 mM sodium phosphate buffer (pH 7.0) with 77 mM sodium chloride and 0.01% BSA, was pre-incubated with 50 µL of the sample in concentrations ranging from between 0.100 μM and 100 μM for 10 min at 37 °C. Subsequently, the assay was initiated by adding 25 µL hyaluronic acid (0.015 % in 300 mM sodium phosphate, pH 5.35) to the incubation mixture and incubated for an additional 30 min at 37 °C. Undigested hyaluronic acid was precipitated with 200 µL acid albumin solution composed of 0.1 % bovine serum albumin in 24 mM sodium acetate and 79 mM acetic acid (pH 3.75). After standing at room temperature for 10 min, the absorbance of the reaction mixture was measured at 600 nm. The absorbance in the absence of enzymes (blank) served as the reference value for maximum absorbance. Sodium aurothiomalate was used as a control inhibitor. Enzyme activity was verified by a control experiment conducted simultaneously, in which the enzyme was pre-incubated with buffer instead, followed by the assay procedures described above. The inhibitory activity of the test compound was determined as follows:

%Inhibition = [1 − (Abs_blank_ − Abs_sample_)/(Abs_blank_ − Abs_control_)] × 100 (4)

where Abs_blank_ is the absorbance of the reaction without enzyme, Abs_sample_ is the absorbance of the extract or standard compound sodium aurothiomalate, and Abs_control_ is the absorbance of the enzyme without inhibitor. The IC_50_ value, which was the sample concentration that inhibited 50% of the enzyme activity, was determined by interpolation from the % hyaluronidase inhibition vs. concentration curve.

#### 3.4.5. Tyrosinase Inhibition Assay

The extracts were assayed by adapting the tyrosinase inhibition method described by Shimizu et al. [30] and modified by Manosroi et al. [31]. In brief, 25 μL of tyrosinase enzyme solution (135 U/mL), 25 μL of ten serial concentrations of the extracts (0.100–100 μM dissolved in 100 mM phosphate buffer, pH 6.8 containing no more than 2.5 % DMSO), and 100 μL phosphate buffer were mixed in a 96-well plate and incubated at 37 °C for 20 min. Then, 50 μL of 1.66 mM of tyrosine solution in 100 mM phosphate buffer, pH 6.8, was added. The enzyme activity was measured at 490 nm every 10 min for 30 min. Kojic acid at 0.100–100 μM was used as a control inhibitor. The experiments were carried out in triplicate. For each concentration, enzyme activity was calculated as a percentage of the velocities compared to that of the assay using buffer without any inhibitor. The IC_50_ value, which was the sample concentration that inhibited 50% of the enzyme activity, was determined by interpolation from the % tyrosinase inhibition vs. concentration curve.

#### 3.4.6. Elastase Inhibition Assay

The extracts were assayed by the method described by Ndlovu et al. [32] with some modifications. In brief, 25 μL of elastase enzyme solution (0.3 U/mL), 50 μL of ten serial concentrations of the extracts or fractions (0.100–100 μM dissolved in 100 mM HEPES buffer, pH 7.5 containing no more than 2.5 % DMSO), and 125 μL HEPES buffer were mixed in a 96-well plate and incubated at room temperature for 20 min. Then, 50 μL of *N*-methoxysuccinyl-Ala-Ala-Pro-Val-p-nitroanilide (1 mM) was added. The enzyme activity was measured at 405 nm in the moment of substrate addition and after 40 min of incubation at 25 °C. *N*-Methoxysuccinyl-Ala-Ala-Pro-chloromethyl ketone at 0.100–100 μM was used as a positive control. The experiments were carried out in triplicate. For each concentration, enzyme activity was calculated as a percentage of the velocities compared to that of the assay, using buffer without any inhibitor. The IC_50_ value, which was the sample concentration that inhibited 50% of the enzyme activity, was determined interpolation from the % elastase inhibition vs. concentration curve.

#### 3.4.7. Collagenase Inhibition Assay

An adaptation of the method of Mandl et al. [33] was used to determine anti-collagenase activity. The following was added to 2 mL test tubes: 25 μL of collagenase solution (0.8 U/mL); 25 μL TES buffer (50 mM) with 0.36 mM calcium chloride, pH 7.4; and 50 μL of test sample or the reference compound EDTA (with concentrations ranging between 0.100 and 100 μM). The tubes were incubated in a water bath at 37 °C for 20 min. Thereafter, 50 μL FALGPA (1 mM) solution was added to the tubes and further incubated for 60 min at 37 °C. To all tubes, 200 μL of a solution containing equal volumes of a 1.6 mg/mL tin chloride (II) solution in 200 mM citrate buffer, pH 5, and 50 mg/mL ninhydrin solution in DMSO were added. All tubes were placed in a water bath (100 °C) for 5 min and left to cool to room temperature before adding 200 μL of 50% isopropanol to each tube. Contents in the tubes were then transferred to respective wells in 96-well plates. Absorbance was detected at 550 nm. EDTA was used as a control inhibitor. Percentage of collagenase inhibition was calculated as

% Collagenase inhibition = [(Abs_control_ − Abs_sample_)]/Abs_control_] × 100 (5)

where Abs_control_ is the absorbance of buffer + collagenase; Abs_eample_ is the absorbance of buffer + collagenase + sample/standard.

All assays were carried out in triplicate and results expressed as IC_50_, i.e., as the concentration yielding 50% of collagenase inhibition, calculated by interpolation from the % collagenase inhibition vs. concentration curve.

### 3.5. Docking Simulations

Molecular docking studies were performed to investigate the binding mode of the more active compounds synthesized and tyrosinase using Autodock Vina v1.1.2 (The Scripps Research Institute, La Jolla, San Diego, CA, USA) [34]. Three-dimensional (3D) structures of tyrosinase (PDB CID: 2Y9X) [35] were downloaded from the RCSB Protein Data Bank (http://www.rcsb.org; accessed on 13 December 2023). For the selected ligands, the structures were downloaded from PubChem (http://pubchem.ncbi.nlm.nih.gov; accessed on 13 December 2023);

The docking input files of both proteins and ligands were generated using AutoDock-Tools v1.5.6 package (ADT; Scripps Research Institute, La Jolla, San Diego, CA, USA) [36]; the proteins were prepared by removing water molecules and merging non-polar hydrogen atoms. The search grid of the key site of tyrosinase was identified as center x: −10.044, center y: −28.706, and center z: −43.443, with dimensions size x: 15, size y: 15, and size z: 15.

Docking accuracy was increased by adjusting the exhaustiveness value to 8. After docking simulations, the best scoring pose was selected using PyMoL v1.7.6 software (DeLano Scientific LLC, Palo Alto, CA, USA) (http://www.pymol.org/, accessed on 13 November 2023). Then, the protein–ligand interactions were visualized using the BIOVIA Discovery Studio Visualizer v21.1.0.0 software (Accelrys, San Diego, CA, USA) (https://discover.3ds.com/discovery-studio-visualizer-download, accessed on 18 November 2023).

### 3.6. Statistical Analysis

A two-way ANOVA, followed by post-hoc HSD Tukey’s test, was used to assess the significant differences between samples in each assayed biological test using the open-source software programs R [37] (4.2.1 for Windows) and RStudio.

## 4. Conclusions

This work described the synthesis of phytol derivatives and evaluated their cosmeceutical potential, focusing on antioxidant, anti-hyperpigmentation, and ECM-degrading enzyme-inhibitory activities. Most of the derivatives were synthesized for the first time (except for **1a**, **1c**, and **1f**), and the anti-aging activities of these compounds were explored for the first time. The data showed that modifications to the phytol backbone significantly altered biological activity, with some derivatives showing improvements in potency. Although the extent varies depending on the specific activity, this work demonstrates that several derivatives exhibit greater activity than phytol in antioxidant assays (**1c**, **1d**, and **1g**), as well as anti-elastase and anti-collagenase activities (**1a** and **1d**). This highlights the potential to modulate activity levels, with clear implications for their cosmeceutical potential.

The best results were obtained for the tyrosinase inhibition assay, particularly by adding a methoxy benzoate group to the phytol backbone (**1d**). This modification significantly enhanced activity, while others, such as 4-chlorobenzoate and heterocyclic groups, decreased potency. This highlights the importance of substitution patterns for optimizing the anti-hyperpigmentation effects of phytol. Molecular docking studies confirmed that the modifications potentiated a stronger interaction between the synthesized compounds and the target enzymes.

In conclusion, this study successfully demonstrated that modifying the phytol structure can significantly enhance both antioxidant and anti-aging activities, leading to the development of multi-target anti-aging compounds for topical applications with greater cosmeceutical potential. Future studies should focus on two points, further refining structural modifications and developing new formulations.

## Figures and Tables

**Table 1:** Summary of the yields obtained on the synthesis of compounds **1a**–**i**.

Phytol Derivatives	Reaction Time (h)	Yield (%)
**1a**	24	87
**1b**	24	91
**1c**	20	5
**1d**	17	79
**1e**	21	69
**1f**	22	89
**1g**	114	25
**1h**	16	85
**1i**	16	90

**Table 2:** ^1^H- and ^13^C-NMR data (ppm) of phytol (**1**).

No	Carbon Type	^1^H (ppm)	^13^C
1	CH_2_	4.11 (dd)	59.2
2	CH	5.38 (m)	123.3
3	C		139.7
4	CH_2_	1.92–2.05 (m)	39.9
5	CH_2_	1.36–1.40 (m)	25.2
6	CH_2_	1.25–1.30 (m)	37.4
7	CH	1.30–1.45 (m)	32.7
8	CH_2_	1.25–1.30 (m)	37.3
9	CH_2_	1.36–1.40 (m)	24.5
10	CH_2_	1.25–1.30 (m)	36.7
11	CH	1.30–1.45 (m)	32.8
12	CH_2_	1.25–1.30 (m)	37.3
13	CH_2_	1.36–1.40 (m)	24.8
14	CH_2_	1.10–1.15 (m)	39.4
15	CH	1.50–1.55 (m)	28.0
16	CH_3_	0.84 (d)	22.6
17	CH_3_	0.84 (d)	22.7
18	CH_3_	0.85 (d)	19.7
19	CH_3_	0.85 (d)	19.7
20	CH_3_	1.64 (s)	16.1

**Table 3:** IC_50_ (µM) of the derivatives against tyrosinase.

Compound	IC_50_ (µM)
**1**	77.47 ± 0.80 ^a^
**1a**	34.73 ± 0.10 ^b^
**1b**	59.62 ± 0.51 ^c^
**1c**	78.96 ± 0.87 ^a^
**1d**	27.94 ± 0.63 ^d^
**1e**	>100 ^e^
**1f**	95.68 ± 0.94 ^f^
**1g**	64.11 ± 0.67 ^g^
**1h**	>100 ^e^
**1i**	>100 ^e^
**Kojic acid**	12.30 ± 0.10 ^h^

Different letters indicate significant differences between IC_50_ values (*p* < 0.05).

**Table 4:** Docking score and binding residues of the different ligands with tyrosinase.

Compound	Binding Score (Kcal/mol)	Binding Residues
**1**	−3.1	His85, His263, Met280
**1a**	−3.8	His65, His85, His 263, Arg268, Met280
**1b**	−3.3	His85, His 263, Arg268, Met280
**1c**	−3.1	His 263, Arg268, Met280
**1d**	−4.1	His65, His85, Asn260, His 263, Arg268, Met280
**Kojic acid**	−5.5	His85, His259, His263, Met280

## Data Availability

The data presented in this study are available in the article.
